# A New Pipe Line

**Published:** 1881-10

**Authors:** 


### A NEW PIPE LINE.

The Buffalo and Rock City Pipe Line Company began to deliver oil at Buffalo August 23. The line of pipe is between sixty-three and sixty-four miles in length and four inches in diameter. Rock City, at its southerly terminus, is an old village near the Pennsylvania State Line, and occupying an elevation 1,900 feet above Buffalo. At this point are situated large iron tanks, with a capacity of 25,000 barrels each, for receiving the oil. There is a pump station supplied with improved triplex pumps for pumping the oil, and smaller pumps for supplying to the boilers. Gas from the surrounding wells is used for fuel. Were it not for the intervening hills and valleys between Rock City and Buffalo, oil could be pumped through the entire line with ordinary pressure. In consequence of the numerous high places that the line passes over, a second or relay station is situated about midway between the termini of the line. From this relay station the oil is taken up as it comes from Rock City and forced to Buffalo. Before starting the oil the line was tested with water; hydraulic gauges were put on the line at various points where pressure would be the heaviest, and also at the pumps. By the use of these gauges the speed of the pumps and the gauge of the tanks were taken at stated times, and a record of the pressure and the duty of the pumps was obtained. A report for one hour showed that the pressure was 325 pounds at Rock City end, 625 at Alleghany, a point 950 feet below. The duty of the pumps was 150 barrels an hour. The pipe was tested to 1,200 pounds. On sending in the oil the pressure at Rock City was 200 pounds and 480 at Alleghany, with the same result on the duty of the pumps, showing that nearly double the amount of oil can be pumped through the line on the same pressure required for water. This is the first independent line organized under the act of 1878. The tanks at Buffalo have a capacity of 148,000 barrels.—*Scientific American.*

The leading article in the July number of the *Princeton Review* contains some interesting facts in relation to the distribution of plants and animals. It cites, as the general consensus of geologists, that from 28,000,000 to 100,000,000 years must have elapsed from the time when the earth was a molten mass to the condition in which we now see it. Within this limit must be placed the creation and distribution of plants and animals. The earth is gradually cooling, and the resultant contraction of the interior of the earth has ridged up and folded the rocks, producing mountain chains. This process began in the earliest geological periods and has been repeated at long intervals, the original lines of elevation and folding guiding those formed in each new movement. Along the ridges thus produced, and in the narrower spaces between them much of the sediment carried by water was laid down. Geology estimates that continents are thus worn down at the rate of about a foot in four or five thousand years, and from the fact that the sedimentary deposits of the several geological formations are estimated at about 70,000 feet in thickness, some idea of the immense periods involved in geological eras may be formed. The changes in the topography of the earth's surface and other causes have made enormous revolutions of climate within comparatively late periods and since the date of the introduction of many existing species of animals and plants. The latest of human period of geology is estimated as between ten and eighty thousand years, and is certainly known to have been preceded by an age of excessively cold climate. Eight or nine causes are supposed to have produced this extreme cold period, which was also characterized by immense changes of the extent and relative levels of sea and land. The article in the *Princeton Review* attributes the climatic changes to changes of the earth's surface and consequent alterations of ocean currents. Thus when the American continent was all submerged except two strips on the east and west connected by a belt of Laurentian land on the north, there was a wide Mediterranean sea produced by the tepid waters of the equatorial current, and it swarmed with corals, of which we know no fewer than 150 species, and with many forms of life peculiar to warm seas. With the subsequent changes of the surface there have been periods of cold, and alternations of climate have resulted in changes of animal and vegetable life. It is claimed that the northern regions have been the birthplace of new forms of land life, and the equable and mild climates of the South have sheltered the older forms of life that have died out in more rigorous situations. To sustain this, and thus account for the distribution of plants and animals without admitting the Darwinian theory of the origin of the species and distribution of organized beings, is the purpose of the article referred to.

